# Pretreatment plasma fibrinogen and serum albumin levels predict therapeutic efficacy of concurrent radiochemotherapy for esophageal squamous cell cancer

**DOI:** 10.3389/fonc.2022.1021214

**Published:** 2022-10-13

**Authors:** Jijin Wang, Di Huang, Yuanyuan Wang, Qianqian Yuan, Xue Chen, Yufeng Cheng

**Affiliations:** ^1^ Department of Radiation Oncology, Sun Yat-sen University Cancer Center, State Key Laboratory of Oncology in South China, Guangzhou, China; ^2^ Department of Radiation Oncology, Qilu Hospital of Shandong University, Jinan, China; ^3^ Department of Oncology, Linyi People’s Hospital, Dezhou, China; ^4^ Department of Oncology, Tengzhou Central People’s Hospital, Zaozhuang, China; ^5^ Department of Minimally Invasion Oncology, Shandong Provincial Hospital Affiliated to Shandong First Medical University, Jinan, China

**Keywords:** FA score, esophageal squamous cell carcinoma (ESCC), therapeutic efficacy, survival, concurrent radiochemotherapy

## Abstract

**Purpose:**

Evidence implies that plasma fibrinogen and serum albumin level (FA score) based on plasma fibrinogen and serum albumin is related to cancer prognosis. However, the association between the FA score and therapeutic efficacy of concurrent radiochemotherapy in esophageal squamous cell carcinoma (ESCC) has not yet been evaluated. This study aimed to assess the role of pretreatment FA score in predicting the therapeutic efficacy of concurrent radiochemotherapy for patients with esophageal squamous cell cancer.

**Methods:**

This retrospective study evaluated 154 patients with ESCC who underwent concurrent radiochemotherapy. Receiver operating characteristic curve (ROC) analysis was used to determine the appropriate cut-off values, and multivariate analysis and Kaplan-Meier curve were used to evaluate prognosis.

**Results:**

FA score was significantly associated with the N stage and M stage (*P* = 0.015 and 0.042, respectively). Chi-square analysis/Fisher’s exact tests revealed a correlation between the FA score and curative effect (*P* < 0.001), and higher FA score was associated with poorer treatment effect. Multivariate analysis indicated that FA score (*P* < 0.001) was predictor of overall survival (OS). Kaplan-Meier curve demonstrated that pretreatment FA score was significantly associated with the OS of ESCC: Patient with higher FA score has lower median OS.

**Conclusions:**

The FA score is a reliable prognostic predictor that could assess the curative effect and OS benefit of concurrent radiochemotherapy in patients with ESCC.

## Introduction

Esophageal cancer is the eighth most common cancer and the sixth leading cause of cancer-related mortality worldwide, with a 5-year survival rate of less than 20% (1). Among esophageal cancer subtypes, esophageal squamous cell carcinoma (ESCC) is the most common (1). Although several treatment modalities have emerged, surgical resection and concurrent radiochemotherapy remain major modalities in the treatment of esophageal cancer. Despite remarkable advances of treatment modalities, the mortality rate of patients with esophageal cancer remains high (2). Effective and easily obtainable biomarkers will be valuable in identifying patients with ESCC who may benefit from surgery or concurrent radiochemotherapy. The pathological TNM is an important prognostic indicator in patients with cancer. However, pretreatment biomarkers are rarely used to predict the prognosis of patients with ESCC, and to our best knowledge, no studies have proposed a prognostic index for patients undergoing concurrent radiochemotherapy.

At present, there were many studies on clinical or basic indicators to predict prognosis (3, 4). Recent reports have utilized hemostatic factors and nutritional status to determine patient prognosis after surgery (5). Hyperfibrinogenemia and decrease albumin levels, which is reflected in the level of plasma fibrinogen and serum albumin (FA) (6–11), have been demonstrated to be related to the malignant behaviors of various types of cancer. The FA score, calculated as the plasma fibrinogen level divided by the serum albumin level, may reflect the prognosis of patients with esophageal cancer after surgery (12). However, its usefulness for predicting the efficacy of concurrent radiochemotherapy in ESCC has not been evaluated. Therefore, our study aimed to assess the usefulness of FA score in predicting the short-term therapeutic benefit of concurrent radiochemotherapy and survival of patients with ESCC. Further investigations were made to confirm the prognostic effect of the nutritional index and immune factors after concurrent radiochemotherapy in patients with ESCC.

## Materials and methods

### Study design and patients

This retrospective study reviewed the data of 154 patients (62 women and 92 men; 101 aged ≥65 years) with ESCC who underwent concurrent radiochemotherapy at Tengzhou Central People’s Hospital between January 2011 and December 2014. The exclusion criteria were as follows: 1) surgical therapy; 2) second primary cancer diagnosed within 5 years after the evaluation of cancer index; 3) history of coagulation disorders or metabolic diseases; 4) missing data on pretreatment plasma fibrinogen and serum albumin levels; and 5) loss to follow-up. The albumin and fibrinogen levels were categorized into two subgroups based on the optimal cutoff values. The FA score was assigned (to patients) based on the ratio of fibrinogen to plasma albumin levels and was divided into three categories.

This study was approved by the Institutional Ethics Committee of Qilu Hospital of Shandong University. All patients provided written informed consent for their information to be stored and used in the hospital database.

### Treatment and follow-up

All the patients received platinum-based concurrent radiochemotherapy with radiotherapy doses of 60–66 Gy. The selection of chemotherapy drugs and adjustments of radiotherapy doses were made according to the clinical stage, physician evaluation of the patient condition, and the preferences of the patients and their families. In the first year after treatment, the patients underwent routine examination of hematuria, liver and kidney function, blood biochemistry, tumor markers, upper gastrointestinal angiography, and computed tomography (CT) examinations every 3 months for the first year, then every 6 months for the second and third years, and every year thereafter, with regular follow-ups until death or loss to follow-up.

### Statistical analyses

The median, mean, and standard deviation of serum fibrinogen and albumin levels were calculated. Their optimal cutoff values were determined by ROC (survival status as the outcome) analysis and used for further FA analysis. The categorical variables were presented as cases (%) and Chi-square tests/Fisher’s exact tests were used for comparing differences among different FA groups. Cox proportional hazards regression model (univariate and multivariate analyses) was used to assess prognostic factors and therapeutic evaluations. The Kaplan-Meier curve and log-rank tests were used to analyze OS. All statistical analyses were performed using IBM SPSS Statistics for Windows (version 23.0; IBM Corp., Armonk, NY, USA). All *P*-values calculated were two-sided and *P*-values less than 0.05 were considered statistically significant.

## Results

### Patient baseline clinical characteristics

There were 62 (40.3%) female and 92 (59.7%) male patients. The baseline patient characteristics are shown in **Table 1**. The median age at diagnosis was 68.5 years; 53 (34.4%) and 101 (65.6%) patients were younger and older than 65 years, respectively. Overall, 116 (75.3%) patients had a history of smoking, while 38 (24.7%) patients had never smoked. There were 1.9%, 20.1%, 59.7%, and 18.2% of patients with clinical stage I, II, III, and IV disease, respectively. The median follow-up duration for the survivors was 36.0 months (range, 3.0-108.0 months).

**Table 1:** Baseline clinical characteristics and FA scores of 154 patients with ESCC.

All patients(N=154)		FA score			*P*-value
		0 (n=15,9.7%)	1 (n=46,29.9%)	2 (n=93,60.4%)	
Sex					0.697
Male	92 (59.7%)	8 (53.3%)	26 (56.5%)	58 (62.4%)
Female	62 (40.3%)	7 (46.7%)	20 (43.5%)	35 (37.6%)
Age, years					0.739
< 65	53 (34.4%)	6 (40%)	14 (30.4%)	33 (35.5%)
≥ 65	101 (65.6%)	9 (60%)	32 (69.6%)	60 (64.5%)
History of smoking					0.501*
Yes	116 (75.3%)	13 (86.7%)	33 (71.7%)	70 (75.3%)
No	38 (24.7%)	2 (13.3%)	13 (28.3%)	23 (24.7%)
T stage					0.134*
T1-2	5 (3.2%)	1 (6.7%)	3 (6.5%)	1 (1.1%)
T3-4	149 (96.8%)	14 (93.3%)	43 (93.5%)	92 (98.9%)
N stage					0.015*
N0	30 (19.5%)	7 (46.7%)	9 (19.6%)	14 (15.1%)
N1-2	124 (80.5%)	8 (53.3%)	37 (80.4%)	79 (84.9%)
M stage					0.042*
M0	124 (80.5%)	13 (86.7%)	42 (91.3%)	69 (74.2%)
M1	30 (19.5%)	2 (13.3%)	4 (8.7%)	24 (25.8%)
TNM stage					0.073*
I	3 (1.9%)	1 (6.7%)	1 (2.2%)	1 (1.1%)
II	31 (20.1%)	7 (46.7%)	6 (13.0%)	18 (19.4%)
III	92 (59.7%)	5 (33.3%)	32 (69.6%)	55 (59.1%)
IV	28 (18.2%)	2 (13.3%)	7 (15.2%)	19 (20.4%)
Albumin (g/L)					
Mean ± SD	39.27 ± 4.62	45 ± 1.12	40.40 ± 4.85	37.79 ± 3.94
Median (range)	39.6 (22.7–49.8)	45 (43.8–47.5)	40.60 (29.6–49.8)	38.5 (22.7–44.3)
Fibrinogen (g/L)					
Mean ± SD	3.21 ± 0.81	2.1 ± 0.34	2.73 ± 0.70	3.62 ± 0.60
Median (range)	3.17 (1.21–5.0)	2.1 (2.21–2.66)	2.56 (1.79–4.76)	3.51 (2.73–5.0)

*Fisher’s exact test.

TNM, tumor, node, and metastases; SD, standard deviation.

### Distributions of pretreatment plasma fibrinogen, serum albumin, and FA score

The median pretreatment serum albumin and plasma fibrinogen levels were 39.6 g/L and 3.17 g/L, respectively (**Table 1**). ROC curves showed that the optimal cut-off levels for serum albumin and plasma fibrinogen were 43.7 g/L and 2.67 g/L, respectively. Based on these cut-off values, the albumin and fibrinogen levels were categorized into two subgroups. The FA score was then divided into three categories based on these cut-off values: (1) a score of 0 indicated a serum albumin level > 43.7 g/L and a plasma fibrinogen level < 2.67 g/L; (2) a score of 1 indicated that either the serum albumin level was < 43.7 g/L or the plasma fibrinogen level was > 2.67 g/L; and (3) a score of 2 indicated a low albumin level (< 43.7 g/L) and a high fibrinogen level (> 2.67 g/L).

### Correlation analysis between pretreatment FA score and clinical characteristics

As shown in **Table 1**, the FA score was divided into three groups based on serum albumin and plasma fibrinogen levels. There were 15 (9.7%), 46 (29.9%), and 93 (60.4%) patients with a score of 0, 1, and 2, respectively. The FA score had no significant relationship with sex, age, smoking history, T stage, or TNM stage, but it was significantly associated with the N stage (*P* = 0.015) and the M stage (*P* = 0.042).

### Short-term efficacy analysis

**Figure 1:**
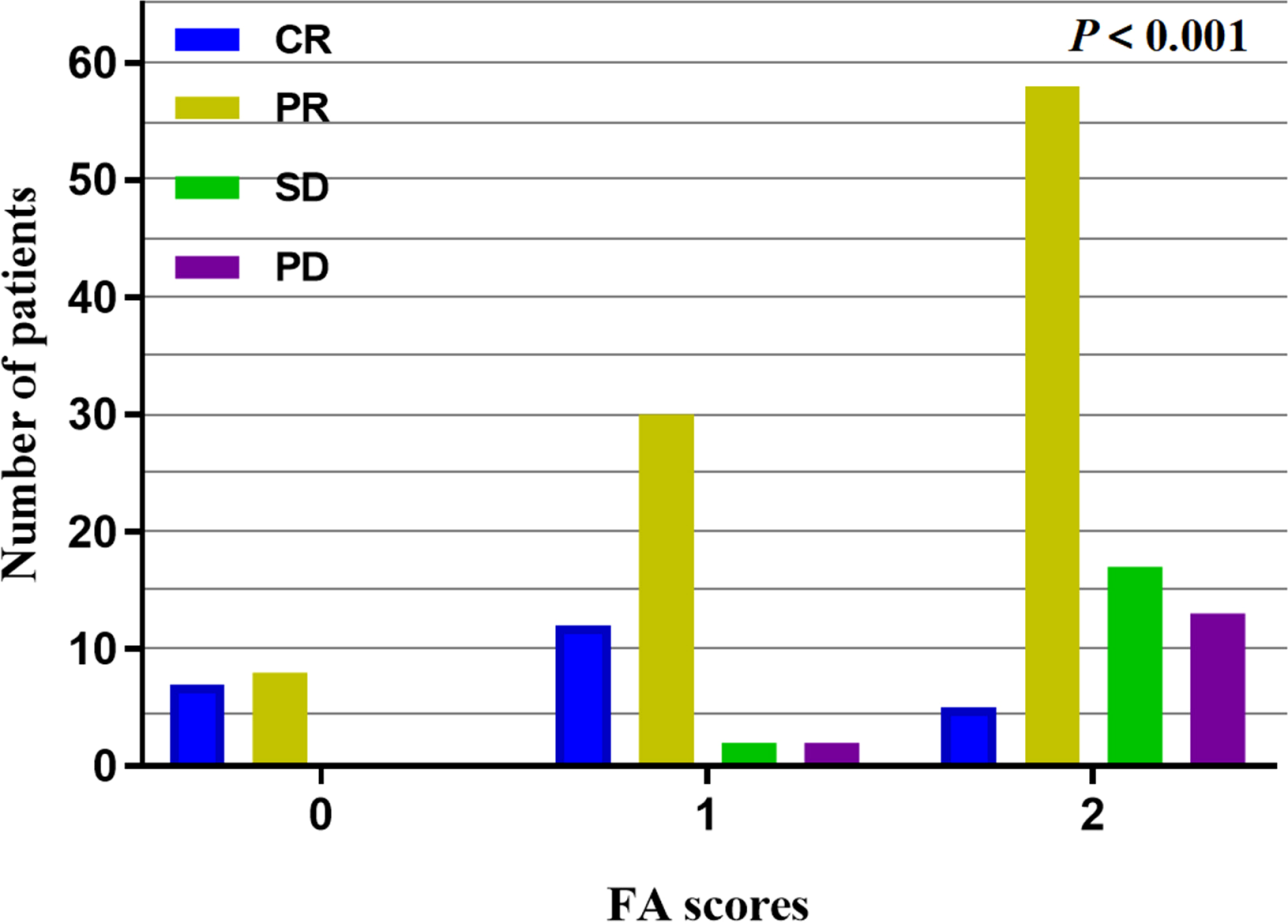
Short-term effect analysis in different FA scores, the higher the FA scores, the lower the ORR with *P* < 0.001.

All 154 patients underwent efficacy evaluation by CT, upper gastrointestinal radiography, and endoscopy 1-3 months after the end of concurrent radiochemotherapy. As shown in **Table 2** and **Figure 1**, 24 (15.6%) patients achieved a complete response (CR), 96 (62.3%) achieved a partial response (PR), 19 (12.3%) patients had stable disease (SD), and 15 (9.7%) patients had progressive disease (PD). The FA score was significantly correlated with the curative effect of radiochemotherapy (*P* < 0.001). Subgroup analysis revealed that the disease control rate (DCR) and objective response rate (ORR) in patients with a FA score of 0 reached 100%, that is, no patients showed disease progression. Meanwhile, 12 and 30 patients with a FA score of 1 achieved CR and PR, respectively, and the ORR was 91.30%. In patients with a FA score of 2, the ORR was 67.74%, but CR was achieved in only 5 patients, accounting for only 5.38% of the total group. Accordingly, significant differences were observed between patients with different FA score, with *P*-values less than 0.001. This indicated that FA score was associated with the short-term effect of radiochemotherapy in patients with ESCC, with higher FA score being associated with a poorer treatment effect.

**Table 2:** Short-term effect analysis in different FA groups.

	Short-term effect				ORR (%)	*P*-value
	CR (n=24,15.6%)	PR (n=96,62.3%)	SD (n=19,12.3%)	PD (n=15,9.7%)		
FA score						< 0.001
0	7 (29.2%)	8 (8.3%)	0	0	100%	
1	12 (50%)	30 (31.3%)	2 (10.5%)	2 (13.3%)	91.3%
2	5 (20.8%)	58 (60.4%)	17 (89.5%)	13 (86.7%)	67.7%	

CR, complete response; PR, partial response; SD, stable disease; PD, progressive disease; ORR, objective response rate.

### Survival analysis

Univariate analysis (**Table 3**) showed no survival differences according to sex, age, history of smoking, T stage, and TNM stage. However, the N stage, M stage, TNM stage, and pretreatment FA score (0, 1, or 2) was significantly associated with OS. Multivariate Cox proportional hazards regression analysis was used to clarify the independent prognostic value of the FA score for OS. Sex, age, smoking, TNM stage, and FA score was included in multivariate analysis. The results revealed that only FA score was predictor of OS (**Table 3**).

**Table 3:** Univariate and multivariate analysis of clinical characteristics related to OS.

Variables	Univariate analysis			Multivariate analysis*		
	HR	95%CI	*P*-value	HR	95%CI	*P*-value
Sex	1.028	0.721–1.466	0.879			
Age	0.962	0.665–1.391	0.835			
Smoking	1.175	0.774–1.782	0.449			
T stage	2.393	0.760–7.539	0.136			
N stage	2.039	1.271–3.271	0.003			
M stage	1.728	1.124–2.656	0.013			
TNM stage I II III IV			0.023			0.164
	Ref.	Ref.		Ref.	Ref.	
	3.508	0.474–25.970	0.219	5.529	0.729–41.929	0.098
	5.472	0.758–39.509	0.092	6.749	0.914–49.863	0.061
	5.474	0.737–40.667	0.097	5.761	0.764–43.421	0.089
FA score 0 1 2			< 0.001			< 0.001
	Ref.	Ref.		Ref.	Ref.	
	4.367	1.811–10.532	0.001	4.359	1.761–10.789	0.001
	7.549	3.244–17.566	< 0.001	7.852	3.283–18.777	< 0.001

*TNM stage was forced into the model and backwards (Wald) of model selection was conducted with other variables. Only the variables that staying in the final model were presented.

CI, confidence interval; HR, hazard ratio.

**Figure 2:**
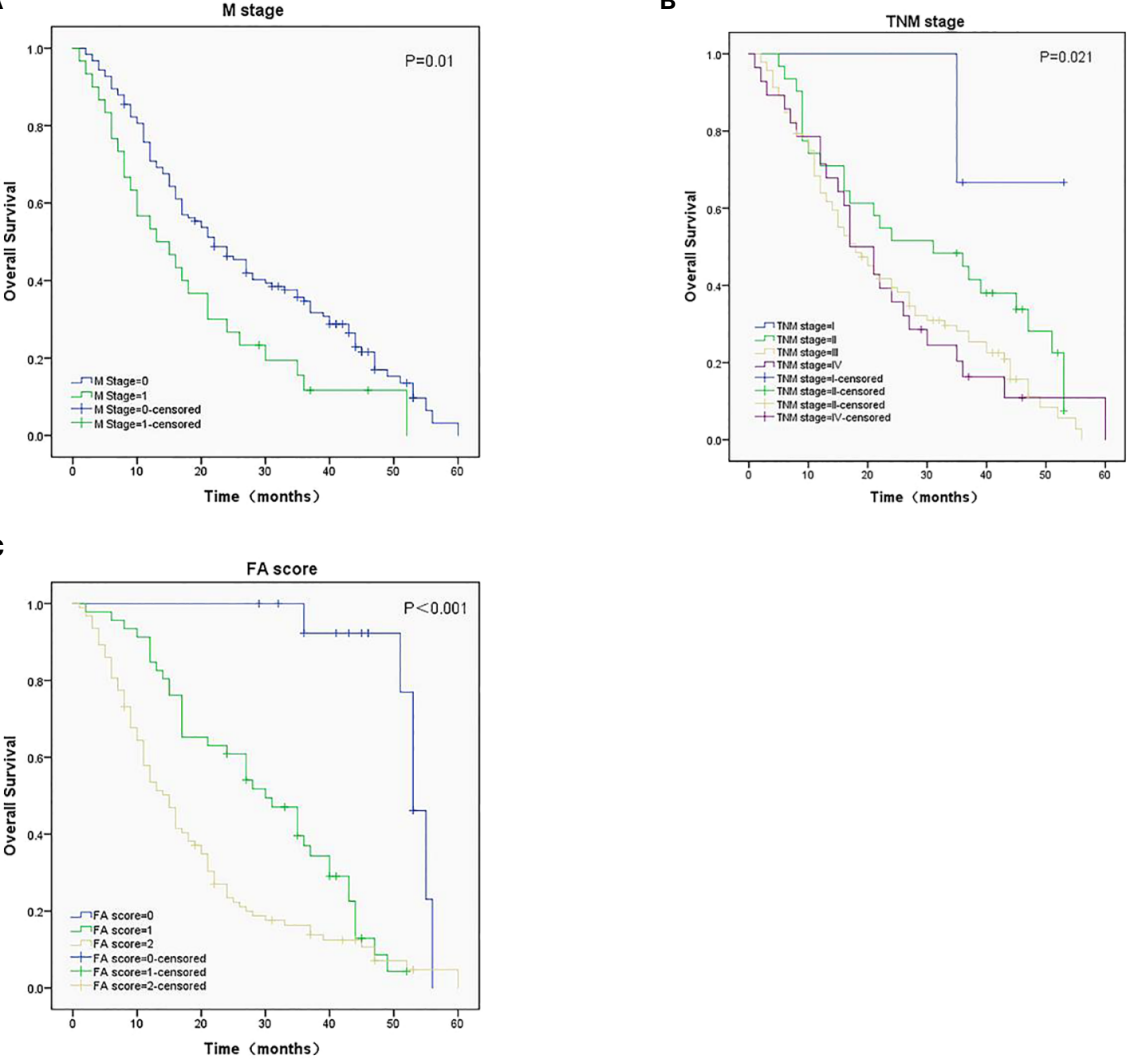
Survival analysis in ESCC patients with different M stage **(A)**, TNM stage **(B)**, and FA score **(C)**, *P*-values were 0.01, 0.021 and < 0.001, respectively.

The median OS (95%CI) were 53 (50-56), 30 (22-38), and 15 (12-18) months for patients with FA score of 0, 1, and 2, respectively. Kaplan-Meier survival analysis showed that the M stage (**Figure 2A**), TNM stage (**Figure 2B**), and FA score (**Figure 2C**) before treatment were significantly associated with OS (*P =* 0.01, 0.021, and < 0.001, respectively). Patient with higher FA score has lower median OS.

## Discussion

Concurrent radiochemotherapy is a major treatment modality for ESCC. As a large proportion of patients lose the opportunity to undergo pathological staging, common prognostic indicators such as the pTNM stage, immunohistochemical features, and other factors cannot be used to evaluate treatment effect. Therefore, an economical, convenient, and objective testing indicator is needed to evaluate the therapeutic effect of concurrent radiochemotherapy in ESCC. The present study shows that the pretreatment FA score is significantly associated with N stage, M stage, and the therapeutic efficacy of radiochemotherapy in ESCC. Higher FA score is associated with a poorer treatment effect and shorter OS. This indicates that the pretreatment FA score may be an important indicator of the therapeutic effect of radiochemotherapy in ESCC.

Fibrinogen is upregulated by cytokines, such as interleukin (IL)-6 in the liver; thus, the plasma fibrinogen level can reflect tumor progression (13). Previous studies have reported that fibrinogen levels are associated with the prognosis of multiple solid tumors (14, 15). In addition, high fibrinogen levels are associated with prognosis and treatment failure in various tumors, including pancreatic, gastric, lung, and colon cancer (16–19). Furthermore, hyperfibrinogenemia has been confirmed to be closely associated with tumor progression (19). In esophageal cancer patients, plasma fibrinogen levels predict postoperative recurrence and survival (14, 20). In addition, fibrinogen is closely associated with metastasis in circulating tumor cells (21, 22). Fibrinogen may play an important role in the development, invasion, metastasis, and recurrence of various tumors. Plasma albumin produced by the liver is one of the indicators used to evaluate nutritional status and is also an independent prognostic indicator in various tumors (23). Studies have shown that hypoalbuminemia is associated with postoperative complications and poor prognosis in several cancers (11, 24, 25).

Matsuda et al. were the first to propose the FA score, calculated as the ratio of fibrinogen to plasma albumin, as a new prognostic marker in cancer (12). Moreover, as a mixed indicator, the FA score can reflect the nutritional status, but also the inflammatory level and coagulation status of patients. A previous study assessed the prognostic impact of FA levels in esophageal cancer, however, the study enrolled patients eligible for surgical treatment (12). This study evaluated the therapeutic effect and OS of patients with non-operative esophageal cancer treated with concurrent radiotherapy and chemotherapy according to the FA score. To our knowledge, no previous study has assessed the CR, PR, SD, and PD after concurrent radiotherapy and chemotherapy in these patients. The tumor microenvironment includes inflammatory cells, immune cells, and cytokines. The interaction of these components in the microenvironment jointly promotes tumor occurrence and development (26). Fibrinogen is an important indicator of systemic inflammation and is closely related to the coagulation system and cytokine production. Fibrinogens regulate inflammatory responses by inducing monocytes to secrete the pro-inflammatory cytokines IL-1, IL-6, and tumor necrosis factor (TNF)-α (27).

Inflammatory responses induced in the tumor microenvironment have been implicated in cancer progression (28). The deposition of fibrinogen promotes the interaction between tumor cells and platelets and accelerates the formation of thrombin, a process that protects tumor cells from natural killer cells (29). In addition, activated coagulation and fibrinolysis systems promote tumor invasion and metastasis. Studies have shown that elevated concentrations of platelets, D-dimer, and antithrombin III are associated with poor prognosis in patients with non-small cell lung cancer (30–32). Tumor cells can secrete fibrinogen, which in turn can promote the proliferation and angiogenesis of tumor cells, thus improving their immune invasion ability (33–35). This process appears to be a vicious cycle. Fibrinogen acting as a scaffold binding, together with fibroblast growth factor–2 and vascular endothelial growth factor, can promote the adhesion, proliferation, and migration of tumor cells (36, 37). The anchoring effect of fibrinogen on growth factors also promotes tumor proliferation and stimulates tumor-related vascular formation (38). Palumb et al. showed decreased incidences of spontaneous hematogenous and lymphatic metastases in tumor-bearing mice after the elimination of circulating fibrinogen (21).

Plasma albumin produced by the liver coordinates transport, maintains intravascular pressure, and clears free radicals (39). It is not only a commonly used indicator to evaluate the nutritional status of the body, but is also used to assess the inflammatory status (24). Malnutrition, which might be reflected by low serum albumin levels, can weaken defense, including the anatomic barriers, cellular and humoral immunity, and phagocyte function (11). Chronic inflammatory response is also associated with tumor proliferation, progression, metastasis, and angiogenesis. Patients with malignant tumors often experience hypoalbuminemia caused by the release of a large number of cytokines, including IL-6 released by tumor cells, which can inhibit the synthesis and secretion of albumin by liver cells. TNF-α, which is related to tumors, is also related to albumin levels. In summary, fibrinogen and plasma albumin play a negative role in the occurrence and development of tumors through several mechanisms, which can also explain the ability of the FA score to be predictive of the treatment efficacy and survival of patients receiving concurrent radiochemotherapy.

In conclusion, a grading system based on the FA score has clinical value for predicting the therapeutic effects of concurrent radiochemotherapy in esophageal cancer. This economical, cost-effective, and practical index can be a useful indicator for planning therapeutic strategies for patients with esophageal cancer undergoing concurrent chemoradiotherapy.

The limitations of this study include its small sample size and single-center retrospective design. In addition, the predictive capability of the FA score for the therapeutic effect of radiochemotherapy was not verified in the validation cohort, thus limiting the clinical value of this study. Moreover, few basic clinical characteristics of the patients could be collected, preventing further subgroup analysis. In addition, the FA score lacks a universal derivation standard and has to be calculated based on specific values for each patient sample. This raises concern of generalizability and is a major limitation in terms of serving as a predictive factor. A multicenter prospective study with a large sample size is warranted to verify the usefulness of the FA score for predicting the therapeutic effect of radiochemotherapy.

## Conclusion

In summary, the FA score can be used to evaluate not only the therapeutic effect (CR, PR, SD, or PD) of concurrent radiotherapy and chemotherapy, but also the OS of patients with ESCC. This indicator has the advantages of being convenient, inexpensive, and noninvasive, thus, it may be a valuable indicator for therapeutic efficacy evaluation and guide clinical practice after prospective studies.

## Data availability statement

The raw data supporting the conclusions of this article will be made available by the authors, without undue reservation.

## Ethics statement

All procedures presented in studies involving human participants were in accordance with the ethical standards of the institutional and/or national research committee and with the 1964 Helsinki Declaration and its later amendments or comparable ethical standards. This study was approved by the Qilu Hospital of Shandong University Human Research Ethics Committee. Written informed consent was obtained from the individual(s) for the publication of any potentially identifiable images or data included in this article.

## Author contributions

YC and XC directed the project, and revised the paper. JW and DH conceptualized and designed the study, analyzed the data, and wrote the paper. YW wrote section of the manuscript. QY analyzed the data. All authors read and approved the final manuscript.

## Funding

This study was supported by the National Natural Science Foundation of China (82172664 and 81972850) and Special Fund for Taishan Scholar Project (ts20190973). Natural Science Foundation of Shandong Province Biomedicine Joint Fund (ZR2021LSW020).

## Conflict of interest

The authors declare that the research was conducted in the absence of any commercial or financial relationships that could be construed as a potential conflict of interest.

## Publisher’s note

All claims expressed in this article are solely those of the authors and do not necessarily represent those of their affiliated organizations, or those of the publisher, the editors and the reviewers. Any product that may be evaluated in this article, or claim that may be made by its manufacturer, is not guaranteed or endorsed by the publisher.

## Abbreviations

TNM, tumor, node, and metastases; ROC, receiver operating characteristic curve; ESCC, esophageal squamous cell carcinoma; FA score, plasma fibrinogen and serum albumin levels; CT, computed tomography; IL, interleukin; OS, overall survival; CR, complete response; PR, partial response; SD, stable disease; PD, progressive disease; TNF, tumor necrosis factor; DCR, disease control rate; ORR, objective response rate.

## References

[1] Napier KJ Scheerer M Misra S. Esophageal cancer: A review of epidemiology, pathogenesis, staging workup and treatment modalities. World J Gastrointest Oncol (2014) 6(5):112–20. doi: 10.4251/wjgo.v6.i5.112 PMC4021327 24834141

[2] Alsop BR Sharma P. Esophageal cancer. Gastroenterol Clin North Am (2016) 45(3):399–412. doi: 10.1016/j.gtc.2016.04.001 27546839

[3] Mohamed AA Omar AAA El-Awady RR Hassan SMA Eitah WMS Ahmed R. Mir-155 and mir-665 role as potential non-invasive biomarkers for hepatocellular carcinoma in Egyptian patients with chronic hepatitis c virus infection. J Transl Int Med (2020) 8(1):32–40. doi: 10.2478/jtim-2020-0006 32435610 PMC7227164

[4] Wang Y Hou K Jin Y Bao B Tang S Qi J. Lung adenocarcinoma-specific three-integrin signature contributes to poor outcomes by metastasis and immune escape pathways. J Transl Int Med (2021) 9(4):249–63. doi: 10.2478/jtim-2021-0046 PMC8802404 35136724

[5] He ZQ Duan H Ke C Zhang XH Guo CCJO. Evaluation of cumulative prognostic score based on pretreatment plasma fibrinogen and serum albumin levels in patients with newly diagnosed high-grade gliomas. Oncotargets (2017) 8(30):49605–14. doi: 10.18632/oncotarget.17849 PMC5564791 28548947

[6] Kijima T Arigami T Uchikado Y Uenosono Y Kita Y Owaki T. Combined fibrinogen and neutrophil-lymphocyte ratio as a prognostic marker of advanced esophageal squamous cell carcinoma. Cancer Sci (2017) 108(2):193–9. doi: 10.1111/cas.13127 PMC5329150 27889946

[7] Qiu J Yu Y Fu Y Ye F Xie X Lu W. Preoperative plasma fibrinogen, platelet count and prognosis in epithelial ovarian cancer. J Obstet Gynaecol Res (2012) 38(4):651–7. doi: 10.1111/j.1447-0756.2011.01780.x 22413879

[8] Wiwanitit V. Re: Preoperative serum albumin as a prognostic factor in patients with upper urinary tract urothelial carcinoma. Int Braz J Urol (2015) 41(4):822. doi: 10.1590/S1677-5538.IBJU.2015.0102 26401881 PMC4757017

[9] Uppal S Al-Niaimi A Rice LW Rose SL Kushner DM Spencer RJ. Preoperative hypoalbuminemia is an independent predictor of poor perioperative outcomes in women undergoing open surgery for gynecologic malignancies. Gynecol Oncol (2013) 131(2):416–22. doi: 10.1016/j.ygyno.2013.08.011 23962700

[10] Lu K Zhu Y Sheng L Liu L Shen L. Wei QJH-g. serum fibrinogen level predicts the therapeutic response and prognosis in patients with locally advanced rectal cancer. Hepatogastroenterology (2011) 58(110-111):1507–10. doi: 10.5754/hge11133 21940318

[11] Ataseven B du Bois A Reinthaller A Traut A Heitz F Aust S. Pre-operative serum albumin is associated with post-operative complication rate and overall survival in patients with epithelial ovarian cancer undergoing cytoreductive surgery. Gynecol Oncol (2015) 138(3):560–5. doi: 10.1016/j.ygyno.2015.07.005 26163893

[12] Matsuda S Takeuchi H Kawakubo H Fukuda K Nakamura R Takahashi T. Cumulative prognostic scores based on plasma fibrinogen and serum albumin levels in esophageal cancer patients treated with transthoracic esophagectomy: Comparison with the Glasgow prognostic score. Ann Surg Oncol (2015) 22(1):302–10. doi: 10.1245/s10434-014-3857-5 24952029

[13] Miki C Konishi N Ojima E Hatada T Inoue Y MJDd K. C-reactive protein as a prognostic variable that reflects uncontrolled up-regulation of the il-1-Il-6 network system in colorectal carcinoma. Dig Dis Sci (2004) 49(6):970–6. doi: 10.1023/B:DDAS.0000034556.48527.6e 15309885

[14] Takeuchi H Ikeuchi S Kitagawa Y Shimada A Oishi T Isobe Y. Pretreatment plasma fibrinogen level correlates with tumor progression and metastasis in patients with squamous cell carcinoma of the esophagus. J Gastroenterol Hepatol (2007) 22(12):2222–7. doi: 10.1111/j.1440-1746.2006.04736.x 18031385

[15] Sun Z-Q Han X-N Wang H-J Tang Y Zhao Z-L Qu Y-L. Prognostic significance of preoperative fibrinogen in patients with colon cancer. World J Gastroenterol (2014) 20(26):8583. doi: 10.3748/wjg.v20.i26.8583 25024612 PMC4093707

[16] Bloomston M Zhou JX Rosemurgy AS Frankel W Muro-Cacho CA Yeatman TJ. Fibrinogen Γ overexpression in pancreatic cancer identified by Large-scale proteomic analysis of serum samples. Cancer Sci (2006) 66(5):2592–9. doi: 10.1158/0008-5472.CAN-05-3659 16510577

[17] Yamashita H Kitayama J Kanno N Yatomi Y Nagawa H. Hyperfibrinogenemia is associated with lymphatic as well as hematogenous metastasis and worse clinical outcome in T2 gastric cancer. BMC Cancer (2006) 6(1):147. doi: 10.1186/1471-2407-6-147 16740157 PMC1501042

[18] Zhao J Zhao M Jin B Yu P Hu X Teng Y. Tumor response and survival in patients with advanced non-Small-Cell lung cancer: The predictive value of chemotherapy-induced changes in fibrinogen. BMC Cancer (2012) 12(1):330. doi: 10.1186/1471-2407-12-330 22852778 PMC3492194

[19] Son H-J Park JW Chang HJ Kim DY Kim BC Kim SY. Preoperative plasma hyperfibrinogenemia is predictive of poor prognosis in patients with nonmetastatic colon cancer. Ann Surg Oncol (2013) 20(9):2908–13. doi: 10.1245/s10434-013-2968-8 23612884

[20] Matsuda S Takeuchi H Fukuda K Nakamura R Takahashi T Wada N. Clinical significance of plasma fibrinogen level as a predictive marker for postoperative recurrence of esophageal squamous cell carcinoma in patients receiving neoadjuvant treatment. Dis Esophagus (2014) 27(7):654–61. doi: 10.1111/dote.12115 23980622

[21] Palumbo JS Kombrinck KW Drew AF Grimes TS Kiser JH Degen JL. Fibrinogen is an important determinant of the metastatic potential of circulating tumor cells. Blood (2000) 96(10):3302–9. doi: 10.1182/blood.V96.10.3302 11071621

[22] Palumbo JS Potter JM Kaplan LS Talmage K Jackson DG Degen JL. Spontaneous hematogenous and lymphatic metastasis, but not primary tumor growth or angiogenesis, is diminished in fibrinogen-deficient mice. Cancer Res. (2002) 62(23):6966–72. 12460914

[23] Gupta D Lis CG. Pretreatment serum albumin as a predictor of cancer survival: A systematic review of the epidemiological literature. Nutr J (2010) 9(1):69. doi: 10.1186/1475-2891-9-69 21176210 PMC3019132

[24] Ku JH Kim M Choi WS Kwak C Kim HH. Preoperative serum albumin as a prognostic factor in patients with upper urinary tract urothelial carcinoma. Int Braz J Urol (2014) 40(6):753–62. doi: 10.1590/S1677-5538.IBJU.2014.06.06 25615244

[25] Geisler JP Linnemeier GC Thomas AJ Manahan KJ. Nutritional assessment using prealbumin as an objective criterion to determine whom should not undergo primary radical cytoreductive surgery for ovarian cancer. Gynecol Oncol (2007) 106(1):128–31. doi: 10.1016/j.ygyno.2007.03.008 17466363

[26] Fridman WH Galon J Dieu-Nosjean M-C Cremer I Fisson S Damotte D. Immune infiltration in human cancer: Prognostic significance and disease control. Cancer Immunol Immunother Springer (2010) . p:1–24. doi: 10.1007/82_2010_46 20512556

[27] Jensen T Kierulf P Sandset PM Klingenberg O Joø GB Godal HC. Fibrinogen and fibrin induce synthesis of proinflammatory cytokines from isolated peripheral blood mononuclear cells. Thromb Haemost (2007) 97(05):822–9. doi: 10.1160/th07-01-0039 17479194

[28] Jennewein C Tran N Paulus P Ellinghaus P Eble JA Zacharowski K. Novel aspects of fibrin (Ogen) fragments during inflammation. Mol Med (2011) 17(5-6):568–73. doi: 10.2119/molmed.2010.00146 PMC3105136 21210072

[29] Zheng S Shen J Jiao Y Liu Y Zhang C Wei M. Platelets and fibrinogen facilitate each other in protecting tumor cells from natural killer cytotoxicity. Cancer Sci (2009) 100(5):859–65. doi: 10.1111/j.1349-7006.2009.01115.x PMC11158185 19302289

[30] Komurcuoglu B Ulusoy S Gayaf M Guler A Ozden EJTJ. Prognostic value of plasma d-dimer levels in lung carcinoma. Tumori (2011) 97(6):743–8. doi: 10.1177/030089161109700611 22322841

[31] Ünsal E Atalay F Atikcan S Yilmaz A. Prognostic significance of hemostatic parameters in patients with lung cancer. Respir Med (2004) 98(2):93–8. doi: 10.1016/j.rmed.2003.07.001 14971870

[32] Barcala FJG Prim JMG Rodriguez MM Fernandez JA Rey MJR Reino AP. Platelet count: Association with prognosis in lung cancer. Med Oncol (2010) 27(2):357–62. doi: 10.1007/s12032-009-9217-9 19381892

[33] Staton CA Brown NJ Lewis CE. The role of fibrinogen and related fragments in tumour angiogenesis and metastasis. Expert Opin Biol Ther (2003) 3(7):1105–20. doi: 10.1517/14712598.3.7.1105 14519075

[34] Steinbrecher KA Horowitz NA Blevins EA Barney KA Shaw MA Harmel-Laws E. Colitis-associated cancer is dependent on the interplay between the hemostatic and inflammatory systems and supported by integrin Amβ2 engagement of fibrinogen. Cancer Sci (2010) 70(7):2634–43. doi: 10.1158/0008-5472.CAN-09-3465 PMC4288842 20233870

[35] Martino MM Briquez PS Ranga A Lutolf MP Hubbell JA. Heparin-binding domain of fibrin (Ogen) binds growth factors and promotes tissue repair when incorporated within a synthetic matrix. Proc Natl Acad Sci U.S.A. (2013) 110(12):4563–8. doi: 10.1073/pnas.1221602110 PMC3607046 23487783

[36] Sahni A Simpson-Haidaris P Sahni S Vaday G Francis CW. Fibrinogen synthesized by cancer cells augments the proliferative effect of fibroblast growth factor-2 (Fgf-2). J Thromb Haemost (2008) 6(1):176–83. doi: 10.1111/j.1538-7836.2007.02808.x 17949478

[37] Sahni A Francis CWJB. Vascular endothelial growth factor binds to fibrinogen and fibrin and stimulates endothelial cell proliferation. Blood (2000) 96(12):3772–8. doi: 10.1182/blood.V96.12.3772 11090059

[38] Witsch E Sela M Yarden YJP. Roles for growth factors in cancer progression. Physiol (Bethesda) (2010) 25(2):85–101. doi: 10.1152/physiol.00045.2009 PMC3062054 20430953

[39] Asher V Lee J Bali A. Preoperative serum albumin is an independent prognostic predictor of survival in ovarian cancer. Med Oncol (2012) 29(3):2005–9. doi: 10.1007/s12032-011-0019-5 21735143

